# Tales of the ER-Golgi Frontier: *Drosophila*-Centric Considerations on Tango1 Function

**DOI:** 10.3389/fcell.2020.619022

**Published:** 2021-01-11

**Authors:** Zhi Feng, Ke Yang, José C. Pastor-Pareja

**Affiliations:** ^1^School of Life Sciences, Tsinghua University, Beijing, China; ^2^Tsinghua-Peking Center for Life Sciences, Beijing, China

**Keywords:** traffic, secretion, ER exit site, COPII, collagen, extracellular matrix

## Abstract

In the secretory pathway, the transfer of cargo from the ER to the Golgi involves dozens of proteins that localize at specific regions of the ER called ER exit sites (ERES), where cargos are concentrated preceding vesicular transport to the Golgi. Despite many years of research, we are missing crucial details of how this highly dynamic ER-Golgi interface is defined, maintained and functions. Mechanisms allowing secretion of large cargos such as the very abundant collagens are also poorly understood. In this context, Tango1, discovered in the fruit fly *Drosophila* and widely conserved in animal evolution, has received a lot of attention in recent years. Tango1, an ERES-localized transmembrane protein, is the single fly member of the MIA/cTAGE family, consisting in humans of TANGO1 and at least 14 different related proteins. After its discovery in flies, a specific role of human TANGO1 in mediating secretion of collagens was reported. However, multiple studies in *Drosophila* have demonstrated that Tango1 is required for secretion of all cargos. At all ERES, through self-interaction and interactions with other proteins, Tango1 aids ERES maintenance and tethering of post-ER membranes. In this review, we discuss discoveries on *Drosophila* Tango1 and put them in relation with research on human MIA/cTAGE proteins. In doing so, we aim to offer an integrated view of Tango1 function and the nature of ER-Golgi transport from an evolutionary perspective.

## Introduction

The endoplasmic reticulum (ER) is the largest and most versatile organelle in eukaryotic cells, capable of generating, contacting and establishing coordinated relations with all other organelles and subcellular compartments. Among such relations, the one between the ER and the Golgi apparatus was the earliest to be recognized (Palade, 1975). ER-Golgi exchanges concentrate at ER exit sites (ERES): specialized ER regions where secretory cargos are collected prior to their trafficking to the Golgi (Bannykh et al., 1996). While dozens of proteins are required for exit of secretory cargo from the ER and entry into the Golgi, few are known to be involved in post-Golgi traffic (Lee et al., 2004; Barlowe and Miller, 2013), a clear indication that ER-to-Golgi cargo transfer at the ER-Golgi interface is the most critical step in secretion. In these same narrow ERES regions, in addition, traffic in the reverse Golgi-to-ER direction takes place as well (Roy Chowdhury et al., 2020). Despite their highly dynamic underlying nature, ERES are overall stable entities, raising the question of how they persist through time. Due to the high concentration of traffic regulators and proteins of the vesicle budding and fusion machineries, it has been postulated that ERES may behave as membrane-less liquid droplets (Hanna et al., 2018). Against this background, Tango1 (Transport and Golgi Organization 1), an ERES-localized protein discovered in the fruit fly *Drosophila*, has captured a great deal of interest for its role in ERES-Golgi coordination.

**FIGURE 1:**
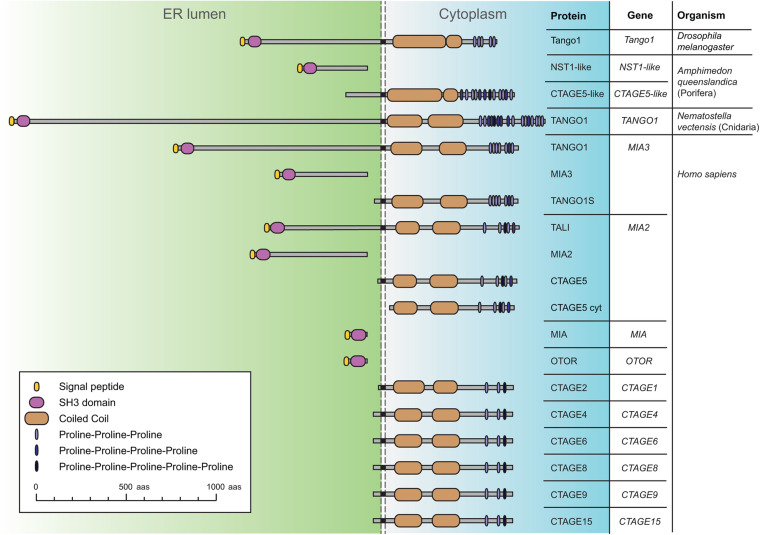
Schematic representation of *Drosophila* Tango1 and proteins of the MIA/cTAGE family, found exclusively in animals. A single representative is found in flies, consisting of an ER-lumenal part, displaying a highly conserved SH3 domain, and a cytoplasmic part, containing coiled coils and a C-terminal proline-rich region. In the sponge *Amphimedon queenslandica*, a representative of the phylum Porifera, the most basal metazoans, ER lumenal and cytoplasmic parts are found as separate proteins. In the sea anemone *Nematostella vectensis*, a cnidarian, a complete TANGO1 protein is found. Human MIA/cTAGE family members include TANGO1 and TANGO1-like (TALI) as complete proteins, but also ER-lumenal and transmembrane cytoplasmic forms, and also at least one predicted completely cytoplasmic CTAGE5 isoform (CTAGE5 cyt). Sequences represented and corresponding RefSeq accession numbers are: Dmel Tango1 (NP_609058.2), Aque NST1-like (XP_019849291.1), Aque CTAGE5-like (XP_019849290.1), Nvec TANGO1 (XP_032221684.1), Hsap TANGO1 (NP_940953.2), Hsap_MIA3 (XP_937620.1), Hsap_TANGO1S (NP_001287796.1), Hsap TALI (NP_001316143.1), Hsap MIA2 (NP_473365.3), Hsap CTAGE5 (NP_976231.1), Hsap CTAGE5cyt (XP_011535087.1), Hsap MIA (NP_001189482.1), Hsap OTOR (NP_064542.1), Hsap CTAGE2 (NP_758441.2), Hsap CTAGE4 (NP_940897.2), Hsap CTAGE6 (NP_848656.2), Hsap CTAGE8 (NP_001265436.1), Hsap CTAGE9 (NP_001139131.1), and Hsap CTAGE15 (NP_001008747.1).

Tango1 is a transmembrane protein of the MIA/cTAGE (Melanoma Inhibitory Activity/Cutaneous T-cell lymphoma-associated antiGEn) family (Figure 1; Usener et al., 2003; Malhotra and Erlmann, 2011). Members of this family were found to be upregulated in melanoma samples before a role in secretion had been described (Bosserhoff et al., 1997; Stoll et al., 2001). The ER-lumenal portions of Tango1 and human TANGO1, encoded by *MIA3*, contain an SH3 domain, typically mediating protein-protein interactions in the cytoplasm, but exceptional in an ER-resident or secreted protein (Stoll and Bosserhoff, 2008). Their cytoplasmic portions, in turn, display conserved regions with no recognizable domains, but presumed to organize as coiled coils, and a C-terminal region that is poorly conserved but proline-rich, with multiple occurrences of consecutive prolines (Saito et al., 2009). Apart from *MIA3*, the human genome possesses 10 additional genes encoding MIA/cTAGE proteins. Among these, TANGO1 and TANGO1-like (TALI), encoded by *MIA2*, most resemble *Drosophila* Tango1. The rest contain exclusively the SH3 domain and ER-lumenal portion, or the cytoplasmic portion, presumably membrane-anchored in most cases.

Yeast has provided for many years a genetically amenable system for studying secretion. Genetic screenings in *Saccharomyces cerevisiae* have identified many secretory genes (Schekman, 2010; Barlowe and Miller, 2013). *Drosophila*, however, is increasingly becoming an excellent alternative to investigate secretion in a higher eukaryote. Most proteins known to play roles in secretion have fly homologues, including COPI and COPII components, Rab GTPases, SNAREs, TRAPP complex, p24 and Golgins (Kondylis and Rabouille, 2009; Muschalik and Munro, 2018). Another advantage of *Drosophila* is the availability of sophisticated genetic tools allowing tagging of endogenous proteins, forward genetic screening and complex loss- and gain-of function experiments that can evaluate physiological readouts and functional significance in real tissue and whole-animal contexts. An apparent difference in secretory pathway organization between *Drosophila* and humans is that in vertebrates ERES-derived vesicles fuse to form an ER-Golgi intermediate compartment (ERGIC) where cargo transits to a single juxtanuclear Golgi ribbon. In flies, like in non-vertebrate animals and plants (Glick and Nakano, 2009; Brandizzi and Barlowe, 2013), Golgi elements remain dispersed throughout the cytoplasm in close proximity to ERES, forming ERES-Golgi units (Ripoche et al., 1994; Kondylis et al., 2011). In the reduced space between *Drosophila* ERES and cis-Golgi, nonetheless, electron microscopy studies have shown the existence of pleiomorphic elements (Kondylis et al., 2005), revealing high complexity of this interface and a possible relation with the vertebrate ERGIC. *Drosophila*, finally, provides a very distinct benefit when compared to mammals in the form of limited gene redundancy. For instance, and in contrast with the enormous complexity of the human family, only one MIA/cTAGE protein exists in *Drosophila*, making flies an ideal system to address its function.

## Discovery of Tango1 in *Drosophila*

**FIGURE 2:**
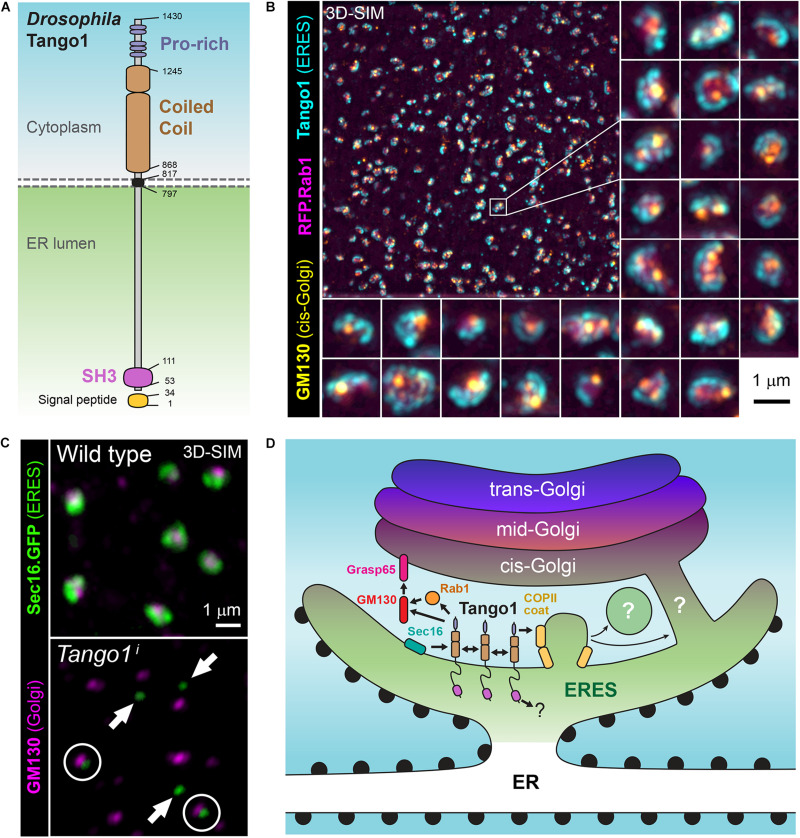
**(A)** Schematic representation of *Drosophila* Tango1. **(B)** Superresolution micrograph (3D-SIM) showing the localization of Tango1 (antibody staining) at ER exit sites (ERES) in a *Drosophila* fat body cell. RFP.Rab1 and cis-Golgi marker GM130 are shown as well. **(C)** Tango1 preserves ERES size and connection ERES-Golgi. *Tango1* knock-down (*Tango1*^*i*^) causes decrease in the size of ERES, marked with Sec16.GFP, and their uncoupling from Golgi (cis-Golgi marker GM130). **(D)** Role of *Drosophila* Tango1 in defining ERES, tethering Golgi membranes and stabilizing the ER-Golgi interface at ERES-Golgi units. Interactions with itself, Grasp65, GM130, Rab1 and Sar1 have been documented (Liu et al., 2017). Additionally, human TANGO1 and other MIA/cTAGE proteins have been collectively found to interact with multiple ERES proteins and ER-Golgi traffic regulators.

A secretory function for *Drosophila* Tango1 (Figure 2A) was first uncovered in a genome-wide RNAi screening for genes required for secretion in *Drosophila* S2 cells (Bard et al., 2006), derived from embryonic macrophages. This screening selected genes for which depletion prevented secretion of ss-HRP (horseradish peroxidase with a signal sequence). A Tango1-V5 fusion, in addition, localized close to mid-Golgi marker ManII-GFP when expressed in S2 cells (Bard et al., 2006), consistent with ERES localization (Figure 2B). Gene hits in this screening that had not been characterized previously in *Drosophila* were named *Tango1* to *Tango14*, for *Transport and Golgi organization*, representing a hypothetical class of animal-specific secretory genes that screenings in yeast could not have uncovered. Most of them retain to this date this *Tango* denomination in their official names, although the only one for which a direct involvement in secretion has been established is *Tango1*. In a second S2 cell RNAi screening, *Tango1* was confirmed to be required for secretion, in this case of a secreted luciferase reporter, and ERES location of its product was observed again (Wendler et al., 2010). RNAi libraries used in the two screenings contained approximately 22,000 double stranded RNAs each and covered close to 100% of *Drosophila* protein-coding genes. Excluding hits nowadays mapping to non-coding or to multiple protein-coding genes, hit lists in the two screenings contained 240 (Bard et al., 2006) and 292 genes (Wendler et al., 2010). Of note, overlap of these two sets of genes, obtained using the same cell line and similar screening systems, was only 24 genes (Ke et al., 2018), which makes it the more remarkable that *Tango1* was selected in both.

## A Collagen Specific Receptor?

After its discovery in flies, human TANGO1 was implicated in the transport of collagens from ERES to Golgi (Saito et al., 2009). At ERES, Golgi-bound, cargo-carrying vesicles are generated by the COPII complex, a set of proteins highly conserved in eukaryotes (Jensen and Schekman, 2011). Structural studies have shown that budding of COPII vesicles is mediated by the assembly of a vesicle-enclosing cage of 60–80 nm in diameter. In animals, many secreted proteins exceed these dimensions and, yet, they are efficiently secreted, raising questions on how this happens and whether specific mechanisms evolved in animals for secretion of large cargos (Fromme and Schekman, 2005). Examples of large secreted proteins include collagens, the main components of animal extracellular matrices, for which trimers assemble in the ER into 300–400 nm long semi-inflexible rods (Canty and Kadler, 2005), but also much larger proteins and protein complexes, such as lipoprotein particles and giant cuticular proteins of insects. Collagen-specific factors creating enlarged transporting vesicles have been postulated, TANGO1 among them (Malhotra and Erlmann, 2015).

TANGO1 silencing in human cells was reported to affect secretion of Collagen VII, but not of other types of collagen or other secreted proteins (Saito et al., 2009; Tanabe et al., 2016). The proline-rich region at the C-terminus of TANGO1 was shown to interact with the COPII coat, while its SH3 domain was described to bind specifically Collagen VII in the ER lumen (Saito et al., 2009). Soon after, however, *MIA3* knock-out mice were revealed to exhibit defects in secretion of multiple collagens (Wilson et al., 2011), while *Drosophila* Tango1 was required for secretion of basement membrane Collagen IV (Pastor-Pareja and Xu, 2011). Activities of TANGO1 in both retarding COPII coat assembly and recruiting ERGIC membranes to nascent vesicles have been proposed as mechanisms by which TANGO1 could mediate formation of enlarged vesicles capable of transporting collagens (Malhotra and Erlmann, 2015; Santos et al., 2015). In this way, TANGO1 would collect collagen at ERES as a specific receptor while at the same time ensuring that a large enough vesicle grows to package it. Additional studies described specific requirements of MIA/cTAGE proteins CTAGE5 and TANGO1S in collagen secretion, and of TALI for secretion of ApoB-containing lipoparticles (Saito et al., 2011, 2014; Maeda et al., 2016; Santos et al., 2016; Tanabe et al., 2016). Other factors reported to affect secretion of collagen specifically were TRAPP complex component Sedlin (Venditti et al., 2012), ubiquitination of Sec31 by ubiquitin ligase KLHL12 (Jin et al., 2012), Syntaxin 18 and SNARE regulator Sly1 (Nogueira et al., 2014). Together with TANGO1 and other MIA/cTAGE proteins, they would be at the core of a collagen-specific, or large protein-specific, secretion pathway.

## Not a Collagen Specific Receptor

Besides its advantages for secretion studies, *Drosophila* is a convenient model to investigate the biology of collagen and the extracellular matrix (Pastor-Pareja, 2020). Compared to the 28 types found in mammals, *Drosophila* possesses a reduced complement of collagens, consisting of basement membrane Collagen IV and Collagen XV/XVIII Multiplexin (Hynes and Zhao, 2000). Multiplexin expression, restricted to heart and nervous system, is dispensable for viability (Meyer and Moussian, 2009). Collagen IV, in contrast, is essential and abundantly present in most tissues. In flies, like in all animals, Collagen IV is the main component of basement membranes: polymers of extracellular matrix proteins that underlie epithelia and provide structural tissue support (Yurchenco, 2011). *Drosophila* Collagen IV is a 450 nm-long heterotrimer composed of α1 chain Collagen at 25C (Cg25C) and α2 chain Viking (Vkg) (Natzle et al., 1982; Lunstrum et al., 1988; Fessler and Fessler, 1989). In the larva, the main source of Collagen IV is fat body adipocytes (Pastor-Pareja and Xu, 2011), known for their role in lipid storage, but also as a very active secretory tissue that produces the serum proteins and clotting factors present in the hemolymph (insect blood). Besides Collagen IV, the other main basement membranes components are Laminin, Nidogen and Perlecan, all conserved from flies to humans as well.

*Drosophila* Tango1 has been repeatedly shown to be required for Collagen IV secretion (Pastor-Pareja and Xu, 2011; Lerner et al., 2013; Isabella and Horne-Badovinac, 2015; Zang et al., 2015; Ke et al., 2018; Sun et al., 2019). However, it was also found that Tango1 loss causes retention of other matrix proteins, such as Perlecan in ovarian follicle cells (Lerner et al., 2013), Papilin in the proventriculus (Zhang et al., 2014), BM-40-SPARC in blood cells (Tiwari et al., 2015) and Laminin in glia (Petley-Ragan et al., 2016). Our laboratory, therefore, decided to carry out a comprehensive analysis of the requirement of Tango1 in secretion (Liu et al., 2017). In this study, Tango1 loss in fat body led to ER retention of not just Collagen IV, but all monitored cargos, including general secretion markers VSVG and secreted GFP (GFP with a signal sequence). Furthermore, Tango1 is expressed in all cell types of the larva and localizes to all ERES, inconsistent with a collagen-specific or large-protein specific role (Liu et al., 2017). Highest expression of Tango1 was found in the salivary gland, a dedicated secretory organ where Tango1 is required for glue secretion (Liu et al., 2017) and secretory genes are highly expressed as a group (Abrams and Andrew, 2005). In all assayed tissues, Tango1 maintains the size and integrity of ERES-Golgi units, as ERES decrease in size and uncouple from Golgi upon Tango1 loss (Figure 2C), whereas Tango1 overexpression, conversely, created more and larger ERES (Liu et al., 2017). Furthermore, supporting an organizing function of Tango1 at the ERES-Golgi interface, the cytoplasmic part of Tango1 could rescue Tango1 loss in the fat body (Liu et al., 2017). Consistent with a general secretion role, Tango1 is needed for secretion of both large and small cargos in terminal cells of the tracheal system (Rios-Barrera et al., 2017), and for secretion of mucins by the male accessory gland and female spermatheca (Reynolds et al., 2019). In all, multiple phenotypic studies demonstrate that Tango1 is not specifically needed for transport of collagens, matrix or large proteins. It is, in contrast, required for general secretion at all ERES, where it maintains ERES size and proximity to Golgi.

The lack of a collagen-specific secretion pathway is further hinted by the results of an RNAi screening in *Drosophila* larval fat body (Ke et al., 2018). This screening, targeting 6,200 genes (about 60% of the protein-coding genome), found 88 genes for which silencing caused intracellular Collagen IV accumulation. Secondary screenings with additional secretory markers revealed that enzymes Prolyl-4-hydroxylase PH4αEFB and Lysyl-oxidase Plod, known to modify collagen post-translationally (Bunt et al., 2011; Pastor-Pareja and Xu, 2011), were the only hits affecting Collagen IV secretion specifically. All other hits affected general secretion, suggesting that collagen secretion does not use devoted mechanisms of vesicular transport. Of the 88 hits in this screening, only 15 were found in the previous S2 cell screenings, with just 7 hits at the intersection of all three: *garz*, *Rab1*, *Use1*, *Slh*, *Syx18*, *Sec23*, and *Sec20* (Bard et al., 2006; Wendler et al., 2010; Ke et al., 2018). The little overlap between the two S2 cell screenings makes it difficult to draw conclusions. Nonetheless, a significant portion of hits in the fat body screening (32 out of 88) encode proteins known to be involved in traffic, including COPII and COPI proteins, SNAREs, TRAPP components, Grasp65, Rab1 and Rab-GTPase regulators (Ke et al., 2018). Remarkably, this screening was able to select known secretion components that neither of the two S2 cell screenings caught, such as Nsf2, Trs23, Trs33, Rep, Grasp65, and Loj (p24), as well as Zn transporter Catsup and SERCA, for which other studies have demonstrated secretory functions (Groth et al., 2013; Suisse and Treisman, 2019). By assaying secretion of an abundant endogenous protein in live animals, this screening may have led to the identification of novel secretory pathway components awaiting characterization. As for collagen secretion, the outcome of the screening points to the absence of a collagen-specific pathway. The requirement of *Drosophila* Tango1 in all secretory contexts, in turn, strongly suggests that human MIA/cTAGE5 proteins act redundantly to maintain ERES and facilitate general secretion. Nonetheless, the possibility remains that the general function of Tango1 in organizing ERES is separate from a specific function in mediating secretion of collagen or other cargos (Rios-Barrera et al., 2017). Indeed, *Drosophila* Tango1 loss seems to affect differentially secretion of Collagen IV when comparing its effects with those of Sec23 and Sar1 knock down on secretion of Collagen IV and secreted GFP (Liu et al., 2017). Whether these differential effects stem from true cargo binding specificity or from size-dependent quantitative differences in transport mode (vesicular vs. tubular carriers?) is currently one of the most pressing questions in the field.

## Mechanisms of Tango1 Function

The function of Tango1 as a stabilizer of the ER-Golgi interface is thought to involve interactions with multiple other proteins (Figure 2D). Through its cytoplasmic part, *Drosophila* Tango1 has been shown to self-interact (Liu et al., 2017). This interaction maps to one of the coiled coil motifs (Reynolds et al., 2019). Importantly, a fully cytoplasmic version of Tango1 correctly localizes to ERES, showing that this portion of the protein is sufficient for localization (Liu et al., 2017). The cytoplasmic portions of human CTAGE5 and TANGO1 had been shown to interact before (Saito et al., 2011), and it is known as well that, similar to *Drosophila* Tango1, human TANGO1 can self-interact (Raote et al., 2018, 2020). Besides self-interaction and interaction with other MIA/cTAGE family members, TANGO1 and related proteins have been shown to bind multiple other proteins present at ERES, such as COPII coat protein SEC23 (Saito et al., 2009; Ma and Goldberg, 2016), SNARE regulator SLY (Nogueira et al., 2014), and ERES determinants SEC16 (Maeda et al., 2017) and SEC12 (Saito et al., 2014). Additionally, *Drosophila* Tango1 co-immunoprecipitates with Rab-GTPase Rab1 and cis-Golgi proteins Grasp65 and GM130 (Liu et al., 2017). Tango1, therefore, through its self-interaction and interaction with other proteins may act as a ER-Golgi interface stabilizer, contributing to concentrating and maintaining localization of all these proteins in a specific region of the ER and to tether post-ER membranes to ERES: Golgi directly in the case of *Drosophila* and ERGIC in vertebrates.

The role of the ER-lumenal part of Tango1, displaying a highly conserved SH3 domain, is still elusive. It has been shown that glycosylation of *Drosophila* Tango1 in the ER lumen prevents cleavage by Furin of this part of the protein (Zhang et al., 2014), suggestive of a proteolytic mechanism in the regulation of Tango1 function for which further details remain to be ascertained. Because Furin localizes to the trans-Golgi network and the endosomal compartments (Thomas, 2002), such cleavage is likely to occur there rather than at ERES, where most Tango1 is found due in part to recycling of the protein from the Golgi (Yuan et al., 2018). A role for the SH3 domain in binding cargos has been proposed. The SH3 domain in human TANGO1 was first reported to bind specifically Collagen VII, but not other collagens (Saito et al., 2009). Later, it was postulated that the SH3 domain could bind HSP47, a collagen chaperone, instead of collagens doing it directly (Ishikawa et al., 2016). Binding of TANGO1 to HSP47, containing an RDEL retrieval signal, would additionally ensure Golgi-to-ER recycling of TANGO1 via KDELR-mediated retrograde transport (Yuan et al., 2018). HSP47, also known as SERPINH1, is a Serpin rather than a typical chaperone. Serpin family members, of which 29 exist in *Drosophila* (Garrett et al., 2009), are highly specific protease inhibitors, some of them secreted proteins themselves, with diverse roles in the immune response. It has been claimed that Spn28F, lacking any RDEL or other putative ER retention motif, is the *Drosophila* homolog of HSP47/SERPINH1 (Sepulveda et al., 2018). However a phylogenetic analysis found no HSP47 orthologs outside of vertebrates (Kumar et al., 2017). A recent study, in addition, found HSP47 was absent from collagen carriers in human cells (Omari et al., 2020). Therefore, the relation between HSP47 and TANGO1, and, more generally, the role of the SH3 domain of Tango1, are still unclear.

## What We Still Do Not Know About Tango1 and Collagen Secretion

Overwhelming evidence in *Drosophila* shows that Tango1 is required for general secretion. However, the question of whether collagen secretion requires any specific factors or uses a mode of ER-Golgi transport essentially different from the one other secreted proteins employ remains open. Besides TANGO1 and MIA/cTAGE proteins, loss of other factors of the general secretion machinery of eukaryotes have been linked to specific defects in collagen secretion in mammals (Boyadjiev et al., 2006; Jin et al., 2012; Venditti et al., 2012; Nogueira et al., 2014). An ER transmembrane protein, TMEM131, has been recently implicated in collagen secretion in *C. elegans*, *Drosophila* and human cells (Zhang et al., 2020), but a requirement for this protein in general secretion or ER homeostasis remains to be tested. It is possible that secretion of collagen and large cargo requires no specific factors, but imposes higher demands on the common secretory machinery. Because collagens are not just very large, but also very abundant (30% of the protein mass of the human body) mild general secretion defects may first become manifest in the form of intracellular collagen retention.

If not a collagen or large cargo receptor, what is the main role of Tango1? Tango1 could have evolved in animals to add stability to ERES, but it is unlikely to be the main upstream factor in an ERES determination cascade. A master role in ERES determination would correspond to Sec16 and Sec12, proteins that, unlike Tango1, are widely conserved from yeast to humans (Glick, 2014; Sprangers and Rabouille, 2015). Recently, it has been shown that phosphorylation of human TANGO1 at its C-terminus regulates ERES disassembly during mitosis (Maeda et al., 2020). However, the putative phosphorylation sites, conserved in vertebrates, do not seem conserved in *Drosophila*. TANGO1 and CTAGE5 have been shown to interact with both SEC16 and SEC12, and thus may cooperate with them in defining ERES through their multiple cytoplasmic interactions (Saito et al., 2014; Maeda et al., 2017). It remains to be seen if any of these interactions is a key high-affinity evolved interaction or rather Tango1 is a generally “sticky” protein ensuring tethering of post-ER membranes and concentration of many different factors at ERES. Interestingly, *C. elegans* has no Tango1 homolog (Erives, 2015), but contains TFG (Witte et al., 2011), absent in flies, which has been proposed to form oligomers that physically join ERES and ERGIC (Johnson et al., 2015; McCaughey et al., 2016). In this evolutionary context, therefore, alternative mechanisms may exist in animal cells to increase stability of ERES and capacity of ER-to-Golgi transport in terms of both cargo size and amount of cargo to be secreted. Unlike TFG, however, Tango1 is a transmembrane protein, and understanding the role of its ER-lumenal SH3 domain, its most conserved part, should be key for fully understanding its function.

Regardless of the specific role of Tango1 in maintaining ERES, the topology of the ER-Golgi interface and the nature of carrier membranes allowing efficient secretion of collagens and other large proteins are hotly debated (Mironov and Beznoussenko, 2019). Transport must involve COPII coated membranes, but these, logically, cannot be regular-sized COPII vesicles. Conflicting studies describe existence or absence of large vesicular carriers transporting collagen (Santos et al., 2015; Gorur et al., 2017; Raote et al., 2017; Yuan et al., 2018; McCaughey et al., 2019; Melville et al., 2019; Matsui et al., 2020; Omari et al., 2020). To be able to visualize collagen transport from ERES in cells, these studies prevent collagen trimerization through ascorbate depletion, followed by ascorbate readministration, which triggers resumption of transport. All these reports, consequently, should be taken with caution, as collagen monomers can still be secreted by cells (Pastor-Pareja and Xu, 2011). What is more, ER-phagy results in such situation (Omari et al., 2018), further complicating interpretation of structures formed under these conditions. The logical alternative to large vesicular carriers as mediators of collagen transport is direct ER-Golgi connection, suggested to occur in yeast (Kurokawa et al., 2014) and plants (daSilva et al., 2004), where ERES and Golgi are closely juxtaposed like in *Drosophila* and, probably, attached physically (Sparkes et al., 2009). ERES-ERGIC contact has been proposed as a transport mechanism in mammals as well (Malhotra and Erlmann, 2015; Raote and Malhotra, 2019). Given the narrow space in which *Drosophila* ERES-Golgi transport takes place, COPII-coated buds may frequently start fusion with cis-Golgi before separating from ERES, thus creating intermittent tubular connections through which all large cargos and most other cargos could reach the Golgi in flies and the ERGIC in vertebrates. Confirmation of this may require electron microscopy analysis of the ER-Golgi frontier, as light microscopy has not been able so far to provide sufficient resolution to settle arguments.

## Author Contributions

All authors wrote the manuscript.

## Conflict of Interest

The authors declare that the research was conducted in the absence of any commercial or financial relationships that could be construed as a potential conflict of interest.
